# False Gastric Diverticulum Arising from the Pylorus Associated with Gastric Outlet Obstruction

**DOI:** 10.1155/2019/3205051

**Published:** 2019-02-14

**Authors:** Umesh Jayarajah, Oshan Basnayake, Pradeep Wijerathne, Jayan Jayasinghe, Nilesh Fernandopulle, Ishan De Zoysa

**Affiliations:** ^1^Professorial Surgical Unit, National Hospital of Sri Lanka, Colombo, Sri Lanka; ^2^Department of Surgery, Faculty of Medicine, University of Colombo, Sri Lanka

## Abstract

A gastric diverticulum is an outpouching from the stomach wall. It is usually seen in the posterior gastric wall and the gastric antrum. Diverticula arising from the pyloric region are extremely rare. A 59-year-old female presented with progressively worsening symptoms of gastric outlet obstruction associated with dyspepsia and vague abdominal pain for 5 years. A large, thin-walled, wide-mouthed, false gastric diverticulum (filled with undigested food) arising from the pylorus associated with gastric outlet stenosis was found by endoscopy and CT imaging. Multiple biopsies from the region excluded a gastric malignancy. A gastrojejunostomy and jejunojejunostomy were performed to bypass the obstruction which successfully relieved the symptoms. This is an unusual site for gastric diverticula, and when associated with gastric outlet obstruction, further distention of the diverticulum may cause more obstruction with worsening symptoms.

## 1. Introduction

A gastric diverticulum is an outpouching from the stomach wall and has similar characteristic diverticula from other parts of the gastrointestinal tract such as the small and large intestines [1]. These are very rare with a prevalence of 0.04% in contrast imaging and 0.01%-0.11% in upper gastrointestinal endoscopies [1]. It occurs usually in the fifth or sixth decades and equally in men and women. There are no pathognomonic clinical symptoms and signs to suggest this condition. Although most patients are asymptomatic, occasionally patients present with abdominal symptoms. These include dyspepsia, vague pain, epigastric fullness, gastrooesophageal reflux disease, and even bleeding or perforation [1, 2]. These are usually seen in the posterior stomach wall and the gastric antrum, and diverticula arising from the pyloric region are extremely rare [1]. We present a case of a large false gastric diverticulum arising from the pylorus associated with gastric outlet obstruction with a brief review of literature.

## 2. Case Presentation

A 59-year-old female presented with symptoms suggestive of gastric outlet obstruction with a background of long-standing dyspepsia. She presented with nonbilious recurrent vomiting of undigested food particles after meals with worsening early satiety for a duration of 5 years. Although she had loss of weight, her appetite was good. She did not have any other medical comorbidities. There was no history suggestive of corrosive injury, gastrointestinal bleeding, obstructive jaundice, or intestinal obstruction. Her body mass index (BMI) was 18.05 kg·m^−2^; however, she did not have any clinical evidence of micronutrient deficiency. Her general and abdominal examination was unremarkable. Her basic biochemistry was normal with a haemoglobin of 11.5 g/dL. Her serum sodium was 132 mmol/L, and potassium was 3.8 mmol/L. Her liver functions were normal with an albumin level of 35 g/L.

**Figure 1:**
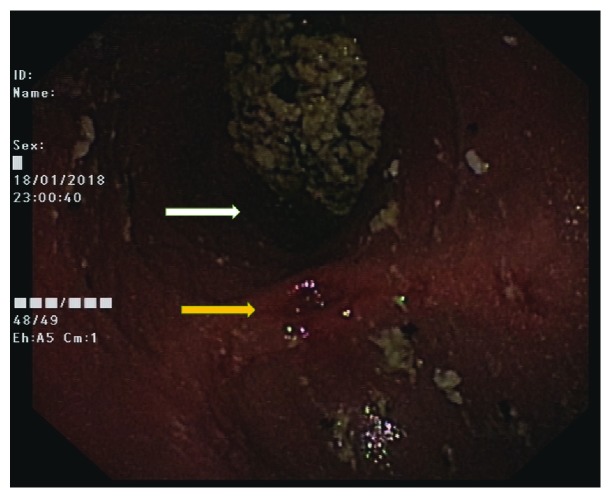
Endoscopic image showing the region of the pylorus. The diverticulum with a wide mouth at the pylorus filled with undigested food is shown by the white arrow, and the stenosed gastric outlet is shown by the yellow arrow.

**Figure 2:**
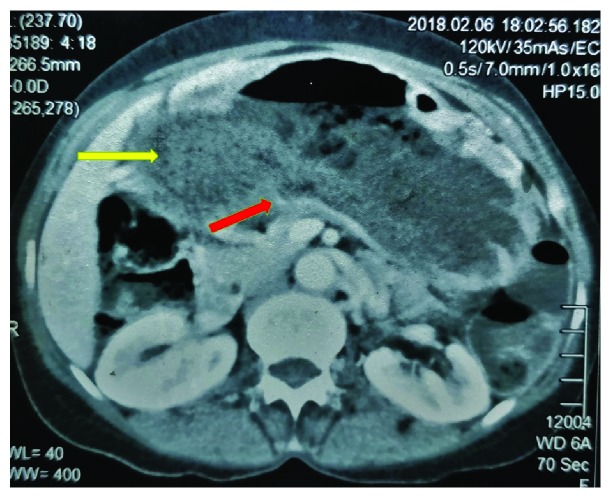
A cross-sectional CECT image showing the level of the pylorus. The false diverticulum arising from the pylorus is shown by the yellow arrow, and the stenosed pyloric canal is shown by the red arrow.

An upper gastrointestinal endoscopy showed a diverticulum with a wide mouth at the pylorus filled with undigested food despite adequate fasting prior to the procedure. The gastric outlet was stenosed, and the scope could not be negotiated beyond it (Figure 1). Multiple biopsies taken from the site were negative for a malignancy and the *Helicobacter pylori* status was negative. Furthermore, there were no visible tumours. Contrast-enhanced computed tomography (CECT) scan showed a distended stomach. There was a large (6 cm × 7 cm × 7 cm), thin-walled outpouching with a wide neck arising from the region of the pylorus filled with gastric contents. The findings were consistent with a false diverticulum arising from the pylorus (Figure 2). The pyloric canal appeared narrowed with no obvious wall thickening or related mass lesions. Passage of oral water contrast medium into the duodenum was noted. The mucosa of the stomach showed normal enhancement following administration of contrast medium. Thus, the CECT and endoscopic findings were in favour of a false diverticulum arising from the pylorus with associated significant stenosis of the pyloric canal.

She underwent an open anterior gastrojejunostomy and jejunojejunostomy. The stomach appeared hypertrophied without any externally visible diverticulum. Pyloroplasty was not possible as there was a large diverticulum at the site. The diverticulum was adherent to the surrounding tissue and resection was difficult. Therefore, it was decided to do a bypass which was the safest procedure for this patient. Her postoperative recovery was unremarkable. Her symptoms were relieved after surgery.

## 3. Discussion

Gastric diverticula are usually small with a diameter of 1-3 cm (range 3-11 cm) and can be classified into true diverticula comprising all gastrointestinal layers and false diverticula which comprise the mucosa and the submucosa [1, 3]. The most common site of gastric diverticulum is the posterior wall of the gastric fundus and antrum [3]. The majority of the true gastric diverticula which are mostly congenital are located in the posterior wall of the fundus of the stomach. False diverticula which are usually acquired were classified as traction or pulsion based on pathogenesis and associated with inflammation or other diseases [1]. Congenital diverticula are believed to originate due to a defect in fusion with the dorsal and the ventral mesenteries with the subsequent formation of diverticulum thus found in the posterior wall. Acquired diverticula are generally found in the antrum and are associated with underlying inflammatory processes such as peptic ulcer disease, malignancy, pancreatitis, and gastric outlet obstruction [1, 4].

The treatment of gastric diverticula depends on the symptom profile of the patient and the comorbid gastrointestinal diseases. Asymptomatic gastric diverticula are best left alone provided that no apparent cause could be identified. In cases of symptomatic diverticula, treatment with proton pump inhibitors has been suggested to relieve the symptoms of gastric diverticula; however, this does not treat the underlying aetiology [5]. Furthermore, in some cases, symptoms of dyspepsia and epigastric pain may be refractory to acid inhibition [6]. Surgical treatment is only recommended for long, symptomatic diverticula refractory to pharmacological therapy and those which are complicated with perforation, bleeding, or suspected of malignancy [1]. Both open and laparoscopic surgical treatments of gastric diverticula have been described with good outcomes [1, 5].

It is important to note that patients with gastric diverticula are symptomatic due to comorbid gastrointestinal diseases and not due to the diverticula per se. Thus, evaluation of these diseases is mandatory before planning treatment. In our patient, the symptoms were due to the gastric outlet obstruction due to the stricture in the pyloric canal which may have been the primary cause for the pulsion-type diverticulum. However, we did not have the facilities to confirm it using electrogastrography monitoring. Furthermore, there were no adhesions with the neighbouring organs to suggest a traction aetiology. However, the unusual site of the diverticulum was seen, i.e., arising from the pylorus. This is significant as further collection of food due to the propulsive action of the hypertrophied gastric musculature causes enlargement of the gastric diverticulum with further narrowing of the pyloric canal leading to a vicious cycle. Associated conditions with gastric diverticula include peptic ulcer disease, malignancy, or pancreatitis [1]. Our patient had no evidence of the above conditions on endoscopy, imaging, and histopathology. Therefore, an aetiology for the pyloric canal stenosis could not be identified in our patient. However, the long-standing symptoms without loss of appetite suggested a benign aetiology. The patient denied any ingestion of corrosive liquids. The CECT and upper gastrointestinal endoscopy were negative for malignancy or peptic ulcer disease. Although less likely, gastric outlet obstruction may be secondary to the false diverticulum arising from the pylorus compressing the pyloric canal and subsequently initiating the viscous cycle.

Therefore, a gastrojejunostomy and jejunojejunostomy were performed to bypass the obstruction and thereby relieve the symptoms. Other minimally invasive treatment modalities for gastric outlet obstruction due to pyloric narrowing include endoscopic dilatation and stenting [7]. Endoscopic dilatation was considered. However, it was a very tight stricture and it was not possible to pass a balloon through it, and even if it was achieved by using a very small-calibre balloon, the benefit is likely to have been temporary.

## 4. Conclusion

We described a patient with a large gastric false diverticulum arising from the pylorus associated with gastric outlet obstruction. This is an unusual site for gastric diverticula and may initiate a vicious cycle of worsening symptoms when associated with a gastric outlet obstruction, requiring surgical intervention.
